# Reversed halo sign in a patient with septic embolism

**DOI:** 10.1590/0037-8682-0630-2023

**Published:** 2024-03-25

**Authors:** Louise Fátima Gomes-Almeida, Nathalia Christina Lopes Flores, Beatriz Rodrigues Bozza, Edson Marchiori

**Affiliations:** 1 Universidade Federal do Rio de Janeiro, Rio de Janeiro, RJ, Brasil.; 2 Universidade Estácio de Sá, Rio de Janeiro, RJ, Brasil.

**FIGURE 1:**
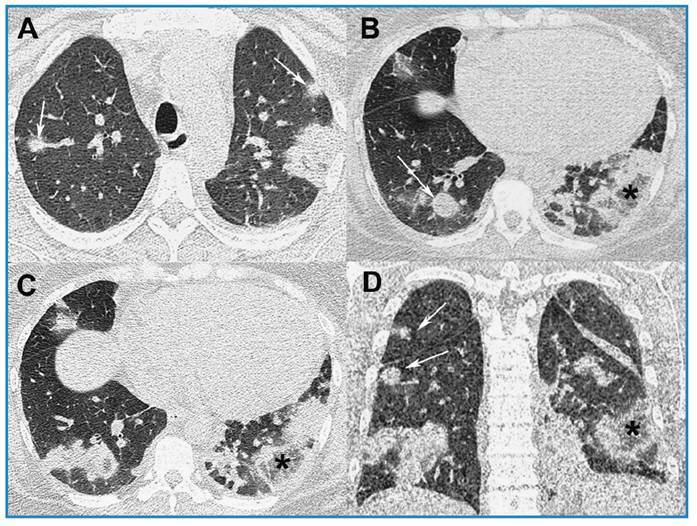
Chest computed tomography images with axial **(A-C)** and coronal **(D)** reconstruction showing multiple ill-defined nodules in both lungs (arrows) and areas of ground-glass opacities and consolidations, some forming reversed halo signs **(asterisks)**.

**FIGURE 2:**
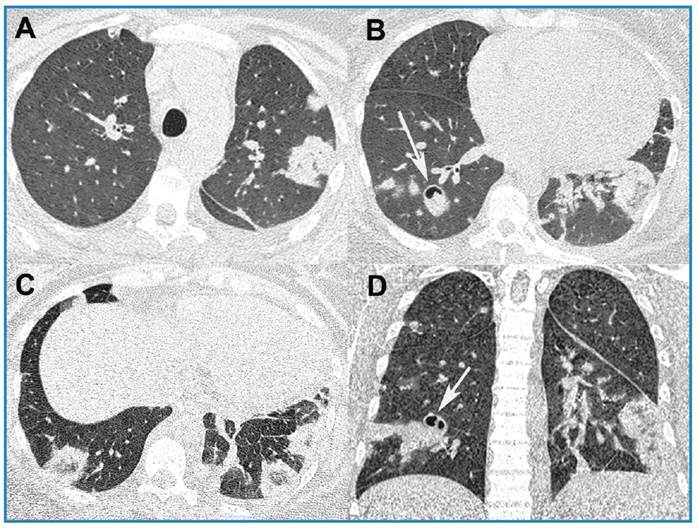
Chest computed tomography images obtained **five** days after the first examination in the same planes. demonstrating partial resolution of lesions, some of which show cavitation **(arrows)**.

A 37-year-old woman with chronic renal failure and mitral insufficiency presented a 15-day history of fever, cough, and dyspnea after hemodialysis. On physical examination, she was eupneic on room air, with 97% O_2_ saturation, and was hemodynamically stable. Laboratory tests revealed leukocytosis (leukocyte count, 18,000/mm^3^), and elevated C-reactive protein (26.3 mg/L). Blood hemocultures were positive for methicillin-sensitive *Staphylococcus aureus*. Infection was observed around the hemodialysis catheter. Unenhanced chest computed tomography (CT) revealed multiple bilateral pulmonary nodules, areas of consolidation, and ground-glass attenuation, some forming reversed halo signs (RHSs; Figure 1). Septic pulmonary embolism (PE) was diagnosed, and antibiotic treatment was initiated, which improved the symptoms. CT examination after five days of treatment initiation revealed partial improvement in opacities, with cavitation of some nodules (Figure 2). The patient was discharged in a stable condition.

CT findings in septic PE mostly include peripheral nodules with or without cavitation^1^. The RHS is a chest CT pattern defined as a focal, rounded area of ground-glass opacity surrounded by a complete or nearly complete ring of consolidation. This sign has been observed in several infectious and non-infectious diseases^2^ ^,^ ^3^, but rarely in cases of septic embolism. However, RHS was recently reported in more than half of intravenous drug users with septic emboli, several of which showed cavitation^3^. Septic PE should thus be considered in the differential diagnosis of patients presenting RHS.
